# Treatment of early non-response in patients with schizophrenia: assessing the efficacy of antipsychotic dose escalation

**DOI:** 10.1186/s12888-015-0629-0

**Published:** 2015-10-31

**Authors:** Antony Loebel, Leslie Citrome, Christoph U. Correll, Jane Xu, Josephine Cucchiaro, John M. Kane

**Affiliations:** Sunovion Pharmaceuticals Inc., Fort Lee, NJ USA; New York Medical College, Valhalla, NY USA; The Zucker Hillside Hospital, Glen Oaks, and the Hofstra North Shore-LIJ School of Medicine, Hempstead, NY USA; ᅟ, 11 Medical Park Drive, Suite 106, Pomona, NY 10970 USA

**Keywords:** Antipsychotic, Dosing, Early responder, Lurasidone, Schizophrenia, Study design

## Abstract

**Background:**

Early non-response to antipsychotic treatment in patients with schizophrenia has been shown in multiple studies to predict poor response at short-term trial endpoint. Therefore, strategies to address the challenge of non-improvement early in the course of treatment are needed. A novel trial design was developed to assess the potential utility of antipsychotic dose escalation in patients with an inadequate initial treatment response. This design was embedded in a study intended to assess the efficacy of low dose lurasidone in patients with schizophrenia. The purpose of this report is to describe the background, rationale and design of this study that included a novel method for the assessment of the potential for dose–response in early non-responding patients with schizophrenia.

**Methods/Design:**

In this 6-week, international, multicenter, double-blind trial, eligible adults with acute schizophrenia were randomized to receive fixed doses of lurasidone 20 mg/day, 80 mg/day (active control), or placebo in a 1:2:1 ratio. Patients initially randomized to lurasidone 80 mg/day who did not have a Positive and Negative Syndrome Scale total score improvement ≥20 % at Week 2 were re-randomized on a 1:1 basis to receive either lurasidone 80 mg/day or lurasidone 160 mg/day for the remainder of the trial. All other groups remained on their initially assigned treatment. The formal primary objective of the study was to evaluate the efficacy of low-dose lurasidone (20 mg/day) compared to placebo; secondary objectives included evaluating the efficacy of lurasidone 80 mg/day versus 160 mg/day in early non-responders, and evaluating the efficacy of lurasidone in all subjects initially randomized to 80 mg/day versus placebo.

**Discussion:**

Since a lack of early improvement predicts poor response to short-term antipsychotic treatment in patients with schizophrenia, several treatment strategies have been proposed to enhance treatment outcome in early non-responders. A novel clinical trial design involving a placebo arm and re-randomization of early non-responders to increased or maintained antipsychotic dose was developed. The study design described in this report provides a robust method to assess the value of antipsychotic dose escalation in patients with schizophrenia who demonstrate poor initial treatment response.

**Trial registration:**

ClinicalTrials.gov NCT01821378; initial registration March 22, 2013

**Electronic supplementary material:**

The online version of this article (doi:10.1186/s12888-015-0629-0) contains supplementary material, which is available to authorized users.

## Background

Treatment guidelines for patients with schizophrenia have typically suggested waiting 4 to 8 weeks to allow adequate time for a patient to respond to an antipsychotic drug prior to switching to another antipsychotic agent [1]. However, in a meta-analysis that included 7450 patients across 42 published studies, reductions in psychopathology were greater in Weeks one and two than in Weeks three and four, and this pattern was present even after the estimated effect of placebo treatment was removed and when results were restricted to the positive symptom subscales of the assessments used [2]. This finding has been replicated by subsequent investigations [3]. Lack of early response to antipsychotics in the acute treatment of schizophrenia has been shown in multiple studies to predict poor response at short-term trial endpoint [1, 4–6]. For example, among 131 patients with schizophrenia receiving open-label fluphenazine 20 mg/day, every patient who experienced an improvement of less than 20 % in Brief Psychiatric Rating Scale (BPRS) total score and 95 % of patients who displayed a reduction of less than 20 % in BPRS thought disturbance factor score following 1 week of treatment were classified as non-responders after 4 weeks of treatment [4]. A pooled analysis was conducted of five randomized, double-blind clinical trials that compared olanzapine to other second-generation antipsychotics in patients with schizophrenia and related disorders. Early response was defined as ≥20 % improvement on the Positive and Negative Syndrome Scale (PANSS) total score at 2 weeks. Conditional probabilities (sensitivity, specificity, positive and negative predictive values) were used to characterize the likelihood of “subsequent response” to treatment (i.e., ≥40 % improvement on the PANSS total score with treatment up to 3 months) [1]. For the receiver operating characteristics curve, the area under the curve was at least 0.75 for all criteria used to assess subsequent response, indicating that the magnitude of early symptom improvement at 2 weeks could predict subsequent response at 3 months. 80 % of non-responders at endpoint were correctly identified as early non-responders at 2 weeks (specificity) and 84 % of early non-responders were non-responders at endpoint (negative predictive value). However, compared to early nonresponse, early response was not as strong a predictor of subsequent response.

When early non-response occurs, potential patient management strategies include increasing the dose of the currently administered antipsychotic medication within the approved dose range or beyond, adding adjunctive medications or switching to another antipsychotic [7]. Another approach to early non-response is to simply wait and continue the initial treatment. However, the chances of achieving a robust antipsychotic response at the starting dose are low in the face of early non-response [1].

Switching to another antipsychotic in the face of early non-response has not been shown to yield substantially better outcomes. In a randomized double-blind, flexible-dosed, 12-week study in patients diagnosed with schizophrenia or schizoaffective disorder, early non-response to risperidone was used to study the potential benefit of switching from risperidone to olanzapine [5]. Early non-response was defined as failure to achieve a ≥20 % improvement on the PANSS total score following 2 weeks of risperidone treatment. Early non-responders were randomized to continue on risperidone 2–6 mg/day or switch to olanzapine 10–20 mg/day for ten additional weeks (early responders remained on risperidone). Early response/non-response was highly predictive of subsequent clinical outcomes. Switching from risperidone in early non-responders to olanzapine at week two resulted in a small, but significantly greater reduction in PANSS total score (−16 points versus −12 points for those staying on risperidone). Of note, the reduction in PANSS total score following the switch to olanzapine was greater among those patients who were still moderately ill at 2 weeks (−22 points versus −16 points for those staying on risperidone) [5]. A robust decrease in the PANSS total score observed amongst early responders to risperidone (−40 points) reinforces the clinical observation that early responders and early non-responders may comprise two distinct patient populations.

Dose escalation in the presence of early non-response has not been well studied. Both switching and dose escalation were examined in an early study in acute schizophrenia where patients received open-label fluphenazine 20 mg/day for 4 weeks [8]. Subjects failing to meet response criteria, which were very stringent (mild or better on each of the four BPRS psychotic items and a rating of much improved or better on the Clinical Global Impressions scale global improvement item) after 4 weeks were then randomized, double-blind, to continue fluphenazine 20 mg/day, dose escalation to fluphenazine 80 mg/day, or switch to haloperidol 20 mg/day [8]. No differences in efficacy were found among these treatment strategies. Dose-escalation in the presence of inadequate response has also been examined in patients with schizophrenia receiving quetiapine [9, 10] and ziprasidone [11]. In these studies, subjects were required to have demonstrated failure on a therapeutic dose in order to be eligible to be randomized to either continue their original dose or to receive a supra-therapeutic dose (quetiapine 1200 mg/day or ziprasidone 320 mg/day depending on the study) under double-blind conditions. In these three studies the higher dose was not superior to the lower. However, none of these four studies used a 2-week decision time point for randomization to dose escalation vs. standard, using instead ≥4 weeks [8–10] or ≥3 weeks [11]. One study [9] used less than minimal improvement (≤15 % reduction on PANSS total sore) as an “early” non-response definition. In addition, these studies used a variety of criteria to assess non-response, some of which were imprecise, such as having at least one moderate positive symptom [8] a relatively high threshold of <30 % PANSS total improvement [10], or “remaining symptomatic” [11]. Moreover, none of these studies included a placebo arm, which may have created a significant expectation bias towards improvement of all subjects.

Lurasidone is a second-generation antipsychotic agent that has received regulatory approval for the treatment of adults with schizophrenia and bipolar depression (both as monotherapy and as adjunct to lithium or valproate) in the US and other countries [12, 13]. The recommended dose range for lurasidone for the treatment of schizophrenia is 40–160 mg/day. There is evidence suggesting a dose response, with lurasidone 160 mg/day resulting in more robust reductions in psychopathology than 80 mg/day in one 6-week acute study [14]. In a post hoc analysis of pooled data from five, 6-week, acute studies of lurasidone, least square mean change-from-baseline in PANSS exhibited a linear trend relative to dose of lurasidone [15]. However, there is a clinical need to understand optimal dosing strategies for lurasidone, including whether dose escalation may be effective in patients with little or no early improvement. As part of the postmarketing commitments made to the US Food and Drug Administration, the utilization of lurasidone in the treatment of adults with schizophrenia with a dose lower than 40 mg (e.g., 20 mg daily) was to be studied through an adequate and well-controlled trial [16]. This report describes the methodology of a novel clinical trial design that in addition to examining the efficacy of lurasidone 20 mg/day, assessed the efficacy and safety of dose escalation in patients with early non-response by re-randomizing early non-responders to either continue 80 mg/day (the initially assigned dose) or to receive 160 mg/day. (Trial Registration: ClinicalTrials.gov NCT01821378).

## Methods/Design

**Fig. 1:**
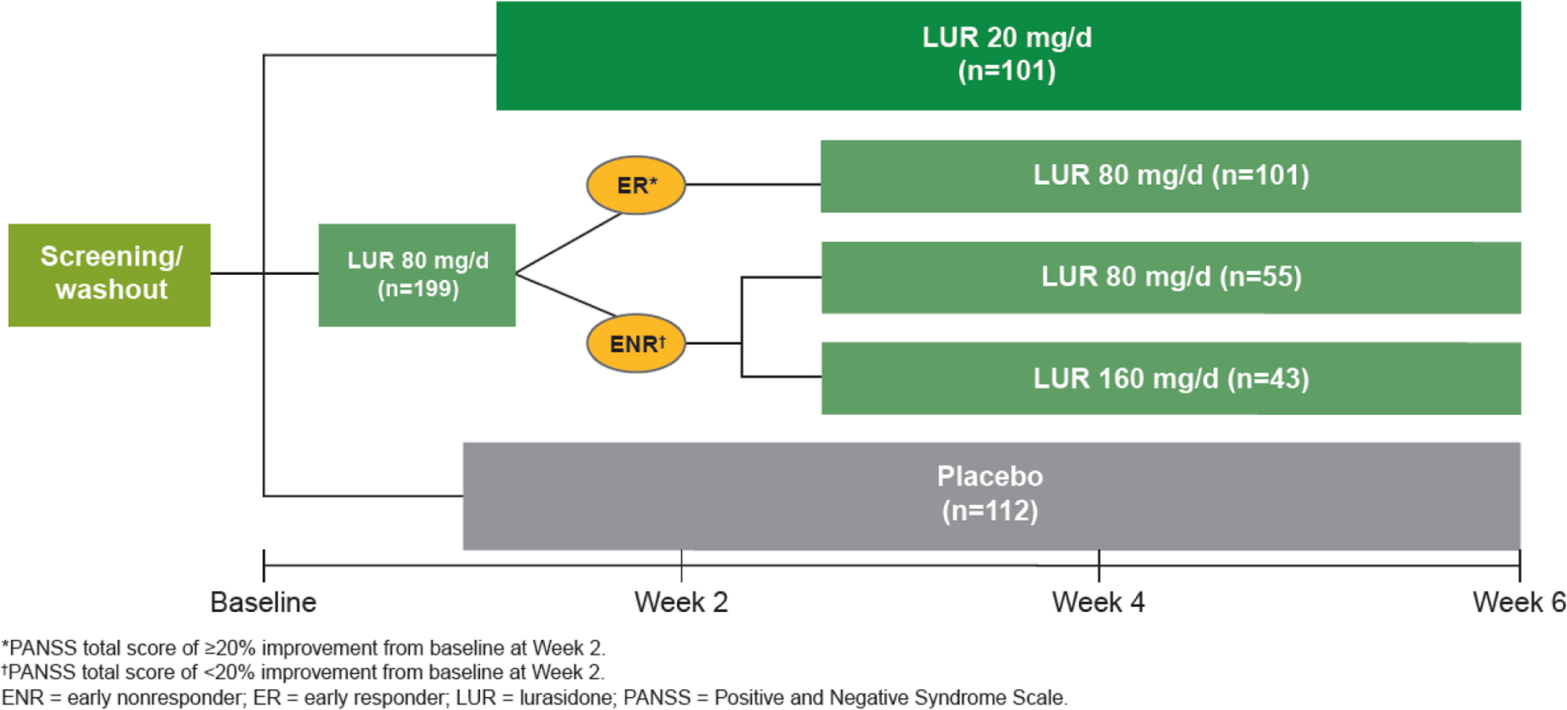
Study design

The study schematic is illustrated in Fig. 1. In this 6-week, international, multi-center, double-blind trial, eligible patients were randomized (via a centralized interactive voice/web response system) to receive fixed doses of lurasidone 20 mg/day, 80 mg/day (active control arm), or placebo on a 1:2:1 ratio. At 2 weeks all subjects were evaluated for early non-response and patients were re-randomized according to the following scheme: subjects assigned to lurasidone 20 mg/day or to placebo continued to receive the same intervention; patients previously randomized to lurasidone 80 mg/day and who had a PANSS improvement ≥20 % at Week two remained on lurasidone 80 mg/day; patients previously randomized to lurasidone 80 mg/day and who had a PANSS improvement < 20 % at Week two were randomized on a 1:1 basis to receive either lurasidone 80 mg/day or lurasidone 160 mg/day for the remainder of the trial.

The study was conducted between May 2013 and June 2014 at 64 sites in the United States, Russia, Romania, Ukraine, Slovakia, and Colombia. The study is undergoing the final data analysis stage.

### Objectives

The formal primary objective of the study was to evaluate the efficacy of low-dose lurasidone (20 mg/day) compared to placebo in patients with an acute exacerbation of schizophrenia; secondary objectives included evaluating the efficacy of lurasidone 80 mg/day versus 160 mg/day in early non-responders, and evaluating the efficacy of lurasidone in all subjects initially randomized to 80 mg/day versus placebo.

### Eligibility criteria

Eligible for participation were men and women between the ages of 18–75 years inclusive, with a diagnosis of DSM-IV-TR schizophrenia, duration of illness ≥ 6 months, PANSS total score ≥ 80 and a PANSS subscale score ≥ 4 (moderate) on two or more of the following PANSS items: delusions, conceptual disorganization, hallucinations, and unusual thought content at screening and baseline, Clinical Global Impressions-Severity of Illness (CGI-S) score of ≥ 4 (moderately ill) at screening and baseline, presence of an acute exacerbation of psychotic symptoms (no longer than 2 months) and marked deterioration of function from baseline (by history) or hospitalized for the purpose of treating an acute psychotic exacerbation for two consecutive weeks or less immediately before screening, and a stable living arrangement. Subjects were also required to be medically stable and those with acute or significant medical conditions were excluded. Subjects who were on stable medication regiments including oral hypoglycemic agents, antihypertensive agents, or thyroid hormone replacement were eligible to participate. Concomitant medications that are CYP3A4 inducers and inhibitors were not permitted. Antidepressants and mood stabilizers (e.g., lithium, divalproex/valproic acid, carbamazepine, etc.) were not permitted. Subjects with treatment resistance to antipsychotic therapy were excluded. The study was approved by an independent ethics committee/institutional review board associated with each investigational site and was conducted in accordance with the International Conference on Harmonisation Good Clinical Practices guidelines and with the ethical principles of the Declaration of Helsinki. A separate file is available with a list of all the names of the independent ethics committees/institutional review boards (Additional file 1). All study participants reviewed and signed an informed consent document explaining study procedures and potential risks before study entry.

### Outcome measures

The PANSS and CGI-S were obtained at screening, baseline (Day 1), and at Days 4, 8, 15, 22, 29, 36 and 43. Outcomes measured included change from baseline in PANSS total score at Week six for each of the lurasidone groups versus placebo. Secondary outcome measures included change in the CGI-S score, PANSS subscale scores, the Montgomery-Asberg Depression Rating Scale (MADRS) total score, and the proportion of subjects who achieved a response, defined as ≥20 % improvement from baseline in PANSS total score. Other measures included adverse events (AEs), AEs leading to discontinuations, and serious AEs, physical examination and laboratory measures, and assessments of extrapyramidal symptoms, suicidality, general health status, functioning, adherence, medication satisfaction, and drop-out risk.

### Study flow

Subjects who met entry criteria (after a 14 day screening phase) entered a 3- to 7-day washout period and remained hospitalized for the duration of the washout. Subjects who demonstrated a decrease (improvement) of ≥ 20 % in the PANSS score between screening and baseline visits or whose PANSS total score fell below 80 at baseline were excluded from this efficacy study. Study medication was administered once daily in the evening with a meal (e.g., dinner) of at least 350 calories or within 30 min of eating. Hospitalization was mandatory through Week three, after which subjects were eligible for hospital discharge if they met specific clinical stability and discharge criteria and could be followed as outpatients for the remainder of the study. Subjects who completed the study and those who discontinued the study early had a follow-up visit 7 days (±2) after the last dose of study medication.

### Data analysis

The study sample size was projected to be 100 subjects in the placebo group, 100 subjects in the lurasidone 20 mg group, and 200 subjects in the lurasidone 80 mg group as determined by two-sample t-tests using nQuery advisor (Version 7.0), based on the treatment effect sizes observed in previous lurasidone studies, to provide a power of 80 % to discern differences between lurasidone 20 mg/day and placebo, as well as between lurasidone 80 mg/day and lurasidone 160 mg/day among the early non-responders, after accounting for potential early drop-outs.

The pre-planned primary efficacy analysis was the examination of the change from baseline in PANSS total score at Week six for lurasidone 20 mg/d versus placebo using a mixed model for repeated measures (MMRM) for the Intent-to-Treat (ITT) population (defined as subjects who were randomized at baseline, received at least one dose of study drug, and had both baseline and at least one post-baseline assessment of the efficacy measure). The MMRM model included treatment, visit, pooled center, baseline scores, and a treatment-by-visit interaction term, using an unstructured covariance matrix. For the key secondary efficacy analysis, the change from baseline to Week 6 in CGI-S score for lurasidone 20 mg versus placebo was analyzed for the ITT population using a similar MMRM model.

The early non-responder ITT population was defined as all subjects who were randomized at Week two, received at least one dose of study medication after the randomization at Week two, and had both a Week two efficacy assessment and at least one efficacy assessment after Week two. Analysis of key secondary efficacy outcomes, change from baseline in PANSS (total score and subscale scores) and CGI-S score for the 80–160 mg early non-responder lurasidone groups was analyzed using MMRM. Change from baseline in MADRS total score was analyzed using analysis of covariance using both observed case and last observation carried forward approaches.

Analyses for PANSS responders were performed using a logistic regression model, which included baseline PANSS total score, pooled center, and treatment.

The safety analyses were conducted using the Safety population (defined as subjects who were randomized and received at least one dose of study medication).

## Discussion

We propose that the study design characteristics reported here provide a robust method to assess the potential for dose–response in early non-responding patients with schizophrenia (or other psychiatric illness).

The key design elements incorporated into this study were: a) initial randomization of patients in double-blind fashion to lurasidone and placebo groups; b) prospective assessment of non-response to study treatment; d) selection of the week two visit to assess level of response to study treatment; e) clear, operationalized definition of non-response; f) re-randomization of early non-responders treated with lurasidone to high dose or standard dose treatment in double-blind fashion and g) assessment of response to lurasidone dose escalation vs continuation of standard dose at the week 6 study visit (4 weeks post-randomization of early non-responders). To our knowledge, the study protocol reported here represents the first study ever conducted that includes these design elements.

Initial lack of improvement is an important predictor of short-term treatment outcome across various psychiatric disorders. This finding has been reported not only for schizophrenia [1], but also in the treatment of major depressive disorder [17, 18] and acute bipolar mania [19]. Several treatment strategies have been proposed to enhance outcome in patients who are early non-responders [7], but few have been rigorously tested.

Using the early antipsychotic response/non-response paradigm, proposed clinical trial designs include an “early responder randomized discontinuation design” where all patients are assigned to the active drug, and only those who had at least a minimal response at 2 weeks are enrolled in a double-blind, placebo-controlled discontinuation trial, enriching the placebo controlled trial portion with true drug responders [20]. In the mirror image “early non-responder randomized dose increase or augmentation design,” early non-responders at 2 weeks are assigned to staying on the medication or going either to a higher dose or an augmentation agent [20]. Our study incorporated the latter methodology as a way of exploring questions regarding dose response in early non-responders to antipsychotic treatment. Importantly, rather than addressing whether or not higher doses of a specific antipsychotic are more efficacious for “all-comers” [21], or in patients not showing clinical response after an adequate trial of 3 weeks or longer [8–11] (using various definitions of non-response that allowed for more than minimal improvement except in one study [9]), this protocol tested whether dose escalation was effective in patients with a clearly inadequate initial treatment response at week two. The population being examined was thus enriched by the specific selection of subjects who prospectively failed to achieve at least minimal response in the first two weeks of the trial. This design permitted a clear demonstration of dose–response in early non-improvers, which may not be evident in studies involving “all comers” or in studies with variable time to early response criteria and imprecise early response definitions. Moreover, previous studies of dose escalation in the face of inadequate response have been confounded by expectation bias that may be present in the absence of a placebo control. The current study minimized this effect by the inclusion of a placebo arm. Whether dose escalation in patients with schizophrenia is an efficacious strategy has important implications regarding optimizing outcomes, maximizing the opportunity for recovery, and managing costs.

The selection of Week two as the point in time to determine non-response was based on prior published work in this area [1–3, 5]. This time point is further supported by naturalistic studies, enhancing the generalizability of this concept [22].

Limitations to the trial included the lack of intermediate doses of lurasidone (i.e., 120 mg/day) to be tested as part of the early non-responder design either as starting or target dose, and that the trial length was only 6 weeks; longer-term outcomes were not tested in this design. Moreover, doses of lurasidone that exceed the maximum recommended amount of 160 mg/day were not tested. A 24-week double-blind randomized controlled trial comparing lurasidone 240 mg/day versus 80 mg/day in treatment resistant schizophrenia or schizoaffective disorder is in progress (NCT01569659). Generalizability of the results of dose escalation of lurasidone in this study to other antipsychotics is not known. SPIRIT checklist is available in a separate file (Additional file 2).

### Summary

The clinical management of early non-response to antipsychotic medication treatment is a common clinical conundrum. This protocol reported here was designed in part to explore the strategy of antipsychotic dose escalation in the presence of early non-response to standard dose treatment. The study design reflected the early non-response paradigm and included blinded re-randomization of early non-responders, as well as an effort to minimize expectation bias and enhance signal detection by the inclusion of a placebo control. In this context, this is the first rigorous study to randomize well-defined, early non-responding patients with schizophrenia to either maintain or escalate an initial dose of antipsychotic treatment in a double-blind fashion, incorporating a placebo-control group.

## Additional files

Additional file 1:
**List of Independent Ethics Committees (IEC) or Institutional Review Boards (IRB).** (DOCX 39 kb) Additional file 2:
**SPIRIT 2013 Checklist: Recommended items to address in a clinical trial protocol and related documents*.** (DOC 119 kb)

## Abbreviations

AE: Adverse event
BPRS: Brief Psychiatric Rating Scale
CGI-S: Clinical Global Impressions-Severity of Illness
DSM-IV-TR: Diagnostic and Statistical Manual of Mental Disorders, Fourth Edition, Text Revision
ITT: Intent-to-Treat
MADRS: Montgomery-Asberg Depression Rating Scale
MMRM: mixed model for repeated measures
PANSS: Positive and Negative Syndrome Scale
