# Supplementary material for: Treatment of early non-response in patients with schizophrenia: assessing the efficacy of antipsychotic dose escalation
Source: BMC Psychiatry. 2015 Oct 31;15:271. doi: 10.1186/s12888-015-0629-0 (PMC4628370; doi:10.1186/s12888-015-0629-0)
Supplement: Additional file 1: — List of Independent Ethics Committees (IEC) or Institutional Review Boards (IRB). (DOCX 39 kb) [file 12888_2015_629_MOESM1_ESM.docx]

List of Independent Ethics Committees (IEC) or Institutional Review Boards (IRB)

1. Schulman Associates IRB, USA
2. UCDS Human Research Protection Program, USA
3. University of Miami - Human Subjects Research Office, USA
4. Via Christi Hospitals Wichita, Inc. Institutional Review Board, USA
5. Comite De Etica En Investigacion de la Empresa Social del Estado Hospital Mental de Antioquia, Colombia
6. Comite de Etica en Investigacion C.E.I Campo Abierto Ltda., Colombia
7. Comisia Nationala de Etica pentru studiu Clinic al Medicamentului, Romania
8. Comisia Nationala de Bioetica a Medicamentului si a Dispozitivelor Medicale, Romania
9. Ethical Council at the Ministry of Health of the Russian Federation, Russia
10. The Independent Interdisciplinary Committee for Ethical Review of Clinical Trials, Russia
11. IEC at St. Petersburg State Public Healthcare Institution "City Psychiatric Hospital No. 4", Russia
12. IEC at FSBI "St. Petersburg Psychoneurological Research Institute n.a. V.M. Bekhterev" of the Ministry of Health of the RF, Russia
13. LIEC at State Budget Healthcare Institution "Chelyabinsk Regional Clinical Hospital", Russia
14. EC at Regional Public Healthcare Institution "Lipetsk Regional Psychoneurological Hospital", Russia
15. Ethics Committee at Municipal Healthcare Institution "City Clinical Hospital #2 named after V.I. Razumovskiy", Russia
16. EC at State Budget Healthcare Institution "Samara Psychiatric Hospital", Russia
17. Ethics Committee at State Public Healthcare Institution of Kemerovo Region "Kemerovo Regional Clinical Psychiatric Hospital", Russia
18. LEC at FSBI “Scientific Research Institute of Mental Health” of Siberian Branch of the Russian Academy of Medical Sciences, Russia
19. Eticka komisia Psychiatricka nemocnica Michalovce, Slovak Republic
20. Eticka komisia Nemocnica s poliklinikou Prievdza so sidlom v Bojniciach, Slovak Republic
21. Eticka komisia Nemocnica s poliklinikou sv. Barbory Roznava, Slovak Republic
22. Eticka komisia Univerzitna nemocnica Bratislava, Nemocnica Ruzinov, Slovak Republic
23. Eticka komisia Vseobecna nemocnica Rimavska Sobota, Slovak Republic
24. Eticka komisia Psychiatricka nemocnica Hronovce, Slovak Republic
25. EC at Communal Treatment and Prevention Institution Regional Clinical Psychiatric Hospital, Ukraine
26. The Ethics Commission at Kyiv City Clinical Psychoneurological Hospital #1, Ukraine
27. The Ethics Commission at the State Treatment and Prevention Institution Central Clinical Hospital of Ukrzaliznytsia, Ukraine
28. The Ethics Commission at Regional Psychoneurological Hospital #3, Ukraine
29. The Ethics Commission at Communal Institution Kherson Regional Psychiatric Hospital of Kherson Regional Council, Ukraine
30. EC at CI O.I. Yushchenko Vinnytsia Regional Psychoneurological Hospital, Ukraine
31. The Ethics Commission at Crimean Republican Institution Clinical Psychiatric Hospital #1, Ukraine
32. The Ethics Commission at Odesa Regional Psychoneurological Dispensary, Ukraine
